# Response of Arctic benthic foraminiferal traits to past environmental changes

**DOI:** 10.1038/s41598-023-47603-w

**Published:** 2023-12-13

**Authors:** Katrine Elnegaard Hansen, Christof Pearce, Marit-Solveig Seidenkrantz

**Affiliations:** 1 Paleoceanography and Paleoclimate Group, Department of Geoscience, Arctic Research Center and iClimate Center, Aarhus University, Aarhus, Denmark; 2 Department of Near Surface Land and Marine Geology, The Geological Survey of Denmark and Greenland (GEUS), Aarhus, Denmark

**Keywords:** Climate-change ecology, Palaeoceanography, Palaeoclimate, Climate sciences, Ecology

## Abstract

The Arctic is subjected to all-encompassing disruptions in marine ecosystems caused by anthropogenic warming. To provide reliable estimates of how future changes will affect the ecosystems, knowledge of Arctic marine ecosystem responses to past environmental variability beyond the instrumental era is essential. Here, we present a novel approach on how to evaluate the state of benthic marine biotic conditions during the deglacial and Holocene period on the Northeast Greenland shelf. Benthic foraminiferal species were assigned traits (e.g., oxygen tolerance, food preferences) aiming to identify past faunal changes as a response to external forcing mechanisms. This approach was applied on sediment cores from offshore Northeast Greenland. We performed numerical rate-of-change detection to determine significant changes in the benthic foraminiferal traits. That way, the significant abrupt trait changes can be assessed across sites, providing a better understanding of the impact of climate drivers on the traits. Our results demonstrate that during the last ~ 14,000 years, bottom water oxygen is the main factor affecting the variability in the benthic foraminiferal faunas in this area. Our results show that significant changes in the traits correspond to drastic climate perturbations. Specifically, the deglacial-Holocene transition and mid-Holocene warm period exhibited significant change, with several trait turnovers.

## Introduction

The Arctic is subjected to an unprecedented rate of temperature increase. In 2022, the temperature in the Arctic exceeded the 1991–2020 average by 1.2 °C, ranking as the fourth warmest year since recordings began in 1950^1^. Including 2022, eight of the warmest years on record have taken place since 2011^1^. This has caused major changes in the distribution and configuration of ocean circulation, ice sheets, sea ice, primary production, biodiversity, and marine ecosystems in the region^2^. The marine food web relies on primary producers, as they occupy the lowest trophic level of the food chain. The primary producers benefit from the release of nutrients from melting sea ice; however, the rapid decline in Arctic sea-ice extent and transitions to more open waters has caused a regime shift in the primary production^3^. Additionally, in connection with the rapid retreat of marine-terminating glaciers in Greenland, elevated meltwater fluxes cause increased stratification in coastal areas, reducing the local primary production and hampering the vertical mixing^4^. On the other hand, marine-terminating glaciers can also induce upwelling and thus increase the surface nutrient supply, which enhances primary production^4^. These changes in primary production have significant impacts on the regional benthic-pelagic coupling, as efficiency of the trophic energy transfer is altered^5,6^. Present and future regime shifts related to climate change will eventually lead to significant alterations of the Arctic marine ecosystems^7,8^. The effect on diversity depends on the individual species’ sensitivity and ability to adapt to changing environments. However, events such as extreme meltwater release and sea-ice retreat can have critical effects on species adaption to new environmental regimes^2,9^. Overall, the observed alterations in biodiversity and marine ecosystems have important consequences for the Arctic food web; but also beyond disrupting food security of native communities and economies that rely on fisheries.

In order to understand the impact of recent and future climate change on Arctic marine ecosystems, knowledge of biotal changes in response to natural climate change prior to the instrumental era is essential. These reconstructions can provide information on the speed of biotic change and long-term consequences of tipping cascades. Here, we assess past changes in benthic foraminiferal traits off NE Greenland in response to environmental variability. Benthic foraminifera are microscopic unicellular organisms, but still comprise a large standing stock in benthic ecosystems. Especially in the Arctic deep seas, benthic foraminifera encompass a large part of the total benthic biomass^10,11^. They generally preserve well in the fossil record and they are sensitive to changes in dissolved oxygen content, temperature, substrate, salinity, and nutrient availability^12,13^. The preferred food sources for most species include diatoms, small chlorophytes, and certain bacteria that sink to the seafloor^12–14^. Thus, they are highly dependent on export of organic particles from the water column, although some species feed on dissolved more refractory organic matter in the sediments^12^. Benthic foraminifera contribute to the biogeochemical cycling in the sediment by feeding on smaller organisms and, although many simply end up in the sediment at the end of their life cycle, others also act as prey for benthic macroorganisms. They are consumed by predators and non-selective deposit feeders such as scaphopod molluscs^14,15^. Hence, they represent a link between lower and higher trophic levels of benthic marine food webs^12,16^. Combined with their relatively short life span this makes them excellent candidates for estimating the state and rapid alterations of marine ecosystems.

Typically, the focus in paleoceanographic studies is on the different individual species and their respective niches, while in ecology, researchers commonly work with so-called trait groups (e.g. species morphology, demands for certain habitat conditions, or tolerance to specific environmental stress factors) consisting of several species to assess ecosystem conditions^17,18^. A trait-based approach allows a more direct assessment of the state of an ecosystem and provides an integrated summary of the full species assemblage. Several studies have already classified and assigned traits to modern plant and phytoplankton communities, aiming to assess and predict overall changes in the communities as a response to external forcing mechanisms^19,20^. Assigning traits to Arctic benthic foraminiferal species is not commonly used in evaluating the speed of change in benthic ecosystems as a response to environmental changes through time. Previous studies have grouped benthic foraminiferal species into different ecological groups to reconstruct environmental changes downcore^21,22^, however they used a different approach to the one we are proposing in this study. Thus, our goals for this study is to reconstruct the paleoenvironmental changes based on the trait approach and to test for significant changes in benthic foraminiferal traits in response to changes in the environment. This will reveal whether trait-based analyses of benthic foraminifera can provide new information on ecosystem response to environmental and climatic change, compared to the traditional species-based reconstructions. We will assess how specific traits (pores, living strategy/mode, substrate preferences, test material, oxygen tolerance, and food preferences) responded to shorter- and longer-lasting climatic perturbations during the last 14,000 years, corresponding to the last deglaciation and the Holocene, a period when significant climatic changes occurred globally^23^. Our study is based on trait analysis of benthic foraminiferal assemblages from eight gravity cores from the northeast Greenland shelf. The Holocene changes in physical properties such as water mass, glacier proximity, and sea-ice cover are known from previous studies, based on X-ray fluorescence core scans, grain size data from marine sediment cores, sea-ice biomarkers, driftwood delivery and, exposure (^10^Be and ^14^C) dates^24–32^. Through statistical methods, we aim to identify and quantify which traits explain most of the variability in the datasets as well as to detect the most significant changes within the benthic foraminiferal traits.

By applying this novel trait-based approach, we aim to capture the key aspects of benthic ecosystem evolution while diminishing the well-known uncertainties involving the correct assessment of the species individual linkages to Arctic environmental conditions.

## Benthic foraminiferal ecology and traits

The term “trait” has been widely used when assessing ecosystem functioning. According to Violle et al. (2007) a trait is a well-defined measurable property of individual organisms (morphological, physiological or phenological), which can be used comparatively across species. Examples of frequently used traits in the marine realm are body size, mobility, and feeding habit^33–35^. The term “trait” is not to be confused with the “traits” often used in genetic studies of foraminifera.

Overall, benthic foraminiferal faunas that flourish in low oxygen conditions are dominated by infaunal species, low diversities, and large standing stocks, potentially linked to low macrofauna presence resulting in lower predation and less competition^12,36,37^. The tests of benthic foraminiferal species living in such conditions are often small, perforated, flattened, elongated, rounded, planispiral, and translucent among others^36–39^ (e.g. *Bolivina pseudopunctata*). The infaunal benthic foraminiferal group is dominated by deposit-feeders capable of ingesting bacteria, degraded phytoplankton remains, and refractory organic matter^40–42^ (e.g. *Melonis barleeanus* and *Lagenammina difflugiformis*). However, some shallow infaunal benthic species have shown to increase their abundance as a response to summer primary production blooms^12^. Likewise, some low-oxygen tolerating species are frequently found dwelling several centimetres into the sediment, if the surface sediments are well-oxygenated^11^. These observations are in line with the conceptual TROX model (TRophic condition and OXygen concentration) which explains microhabitat distribution of benthic foraminifera^43^.

Well-oxygenated marine sediments are primarily occupied by epifaunal species with trochospiral tests (e.g. *Cibicides lobatulus* and *Epistominella arctica*) or milioline coiling (e.g. *Miliolinella subrotunda* and *Triloculina tricarinata*), benefitting from high fluxes of fresh phytoplankton particles^12,44^. In the Greenland-Norwegian Sea, high abundance of epibenthic species are linked to primary productivity blooms at the marginal ice-sea zone from late May to September^44^. Here, the benthic species rapidly utilize the settling of planktonic particles and show high metabolic activity with food vacuoles full of diatoms and dinoflagellates^12,44^.

Research also shows a link between the pore surface area of epifaunal foraminiferal tests and dissolved oxygen concentrations^45–48^. A strong negative logarithmic correlation between pore surface area and bottom-water oxygen concentrations were observed, implicating the potential of reconstructing deep sea oxygen levels and redox conditions based on pore surface area of fossil epifaunal benthic foraminifera^45^. Pore traits were only assigned to calcareous species in our study, since agglutinated foraminifera generally do not have pores and little knowledge exist on the perforation of Arctic agglutinated benthic foraminifera.

Some benthic foraminiferal species tolerate anoxic and sulfidic conditions^49–51^. Some genera (e.g., *Bolivina* and *Stainforthia*) do not only tolerate these harsh conditions; anoxia appears to stimulate the species cellular activity^51^. They are even capable of conducting test calcification during these anaerobic circumstances^46,52^. According to Van der Zwaan et al. (1999) a link between oxygen and substrate preferences has been established for certain species; some oxygen deficiency tolerant species typically reside in muddy substrates, as the oxygen penetration depth is dependent on the sediments grain size distribution. Coarse, sandy substrates have deeper oxygen penetration depths as opposed to clayey muddy sea floors^53^.

The primary food source for benthos comprise the organic fraction produced by pelagic production^12–14^. In the Arctic and Subarctic seas, the flux of these organic food particles from the surface to the bottom waters is short and intensive, followed by longer periods of starvation^12^. Some opportunistic benthic foraminiferal species appear to take advantage of these short lived organic material fluxes, often resulting in low diversity faunas^14,53^. Further information on the traits of various species is provided in Supplementary Table S2.

## Regional settings

### Modern oceanography, sea-ice configuration and surface water productivity

**Figure 1:**
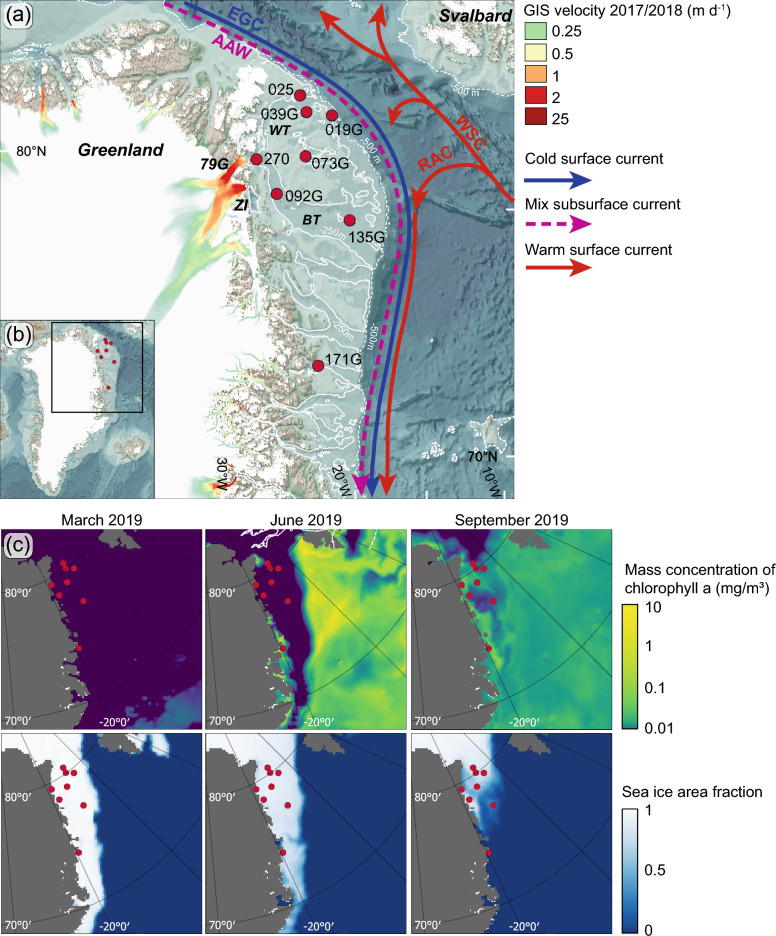
Study area. (**a**) Map of Northeast Greenland showing the main surface and subsurface ocean currents (bold and stippled lines, respectively) and core sites included in this study (for details on core sites see Table 1). The bathymetry is derived from GEBCO^98^. Greenland Ice Stream velocity is derived from Sentinel-SAR data gathered from December 28, 2017 to February 28, 2018^99^ (**b**) Rectangle outlining map (**a**). (**c**) Upper panels are showing satellite based snapshots of chlorophyll *a* mass concentrations on the 15th of March, the 15th of June and 15th of September 2019 generated using the EU Copernicus Marine Environment Monitoring Service^101–103^. Lower panels are showing satellite observation of sea-ice area fraction for the same months in 2019 generated using the EU Copernicus Marine Environment Monitoring Service^100^. *WT* = Westwind Trough, *BT* = Belgica Trough, *79G* = Nioghalvfjerdsfjorden Glacier, *ZI* = Zachariæ Isstrøm Glacier, *RAC* = Return Atlantic Current, *WSC* = West Spitsbergen Current, *EGC* = East Greenland Current, *AAW* = Arctic Atlantic Water.

The Northeast Greenland shelf is influenced by meltwater outflows from drainage of the Northeast Greenland Ice Stream (NEGIS), which ends in three large marine terminating glaciers (Fig. 1). Combined, they drain approximately 12% of the Greenland Ice Sheet^54^. In the subsurface on the Northeast Greenland shelf, Atlantic water masses emanate from the northward flowing West Spitsbergen Current (WSC) (Fig. 1). When the WSC reaches 76–81°N, half of the WSC deflects westward across the Fram Strait transforming into the intermediate and shelf bound Return Atlantic Current (RAC)^55,56^. The other half of the WSC continues northward into the Arctic Ocean, where it recirculates^57,58^. This makes it both colder and fresher before returning to the Fram Strait now denoted Arctic Atlantic Water (AAW)^59,60^. The surface waters on the Northeast Greenland Shelf are comprised by Polar Water in the upper part of the southward flowing East Greenland Current (EGC), which flows along the shelf and is comprised by outflow water masses from the Arctic Ocean, drift ice, and meltwater^61,62^. The Polar Water is colder, and slightly fresher and more oxygen rich than the deeper Atlantic Water masses^58^.

The sea-ice configuration on the northeast Greenland shelf is highly controlled by the drift ice supply from the Arctic Ocean, conveyed by the Transpolar Drift and the EGC^63^. In winter, the majority of the shelf is covered in sea ice, in contrast to the summer months where the sea-ice extent is confined to the northernmost parts of the shelf (Fig. 1). Occasionally in summer, an area at 79° N remains ice-free, which relates to the seasonal opening of the coastal latent heat Northeast Water Polynya (NEW Polynya)^64,65^. Further south at Young Sound (74–75° N), the latent heat Sirius Water Polynya starts forming in winter and remains open until May^66^. When the polynyas are fully established, marine life flourishes, benefitting from the nutrients released by the melting sea-ice edge. In the northwestern Fram Strait region, the highest concentrations of chlorophyll *a* are linked to the NEW Polynya and the marginal sea-ice zone^67^. Consequently, polynyas are of crucial importance for high latitude marine ecosystems and act as carbon sinks^68^. Variations in light availability caused by large seasonal contrasts in both sunlight and sea-ice distribution play a pivotal role on primary production in the western Fram Strait^69^. Concurrently, surface freshening caused by melting of sea ice and elevated glacial meltwater flows alter the stratification and nutrient supply and with it the primary production, the marine food webs and the carbon cycle in Greenland coastal areas^4^. Moreover, the Polar Front coinciding with the marginal sea-ice zone, also serves as an area of enhanced phytoplankton productivity^67^. An extensive sea-ice cover is also regarded as playing a pivotal role in the regulation of bottom water oxygen content as it blocks the air-sea gas exchange^70^. Conversely, the loss of sea ice may foster greater local primary productivity, resulting in organic matter reaching the sea floor and subsequent aerobic microbial decomposition. This process could potentially lead to localized oxygen depletion in sediments and bottom waters^70^. The loss of sea ice is also often associated with rising sea surface temperatures in the Arctic. As bottom waters warm, they can hold less dissolved oxygen^71^. This temperature-driven reduction in oxygen solubility can lead to lower oxygen concentrations in the deep waters^70^. Additionally, when sea ice melts or decreases in extent, it can cause increased stratification in the water column. This stratification can result in reduced vertical mixing of water masses, which, in turn, limits the exchange of oxygen between surface and deeper waters^72^.

### The deglaciation of the Northeast Greenland shelf

The retreat timing and pattern of the Greenland Ice Sheet (GIS) on the Northeast Greenland shelf region is still being studied, but it is clear that the timing of the retreat varied spatially across the Northeast Greenland shelf^24–27,73–75^. Based on the multiproxy record of core DA17-NG-ST01-019G (hereafter 019G; Fig. 1; Table 1) marine conditions were established already at 25.5 cal ka near the shelf edge at 79.4° N (Fig. 1; Table 1)^24^. The radiocarbon dates and lithology of core DA17-NG-ST14-171G (hereafter 171G; Fig. 1; Table 1) near the southern end of the NE Greenland shelf suggest that the GIS was already positioned landward of this core site by 12.2 cal ka BP^28^. Moreover, the inner Norske Trough (Fig. 1) was free from grounded ice by at least c. 13.4 cal ka BP^26^. Further north, in the outer Westwind Trough, NEGIS grounded ice had retreated by c. 13.3 cal ka BP^27^. A multi-proxy analysis of core DA17-NG-ST08-092G (092G) indicate the presence of a floating ice shelf at the inner Norske Trough area during the end of the last glacial^26^, whereas sedimentological characteristics of core DA17-NG-ST03-039G (039G) point to distal glaciomarine conditions from 13.3 to 11 cal ka BP^27^. Further south, a floating ice shelf was located close to core site 171G, according to the lithological features of the basal sedimentary unit^28^.

**Table 1:** Overview of sediment cores included in the study. The oldest and youngest dates are from radiocarbon dating based age models, see original publications for more information on age models.

Name	Short name	Latitude	Longitude	Water depth (m)	Core length (m)	Oldest and youngest modelled median dates (cal yr BP)	References
DA17-NG-ST03-039	039G	80.037	−8.923	390.6	3.20	13269–3922	^27^
DA17-NG-ST08-092G	092G	78.501	−17.279	582.9	5.85	13303–589	^26^
DA17-NG-ST7-73G	73G	79.068	−11.903	385	4.10	9361–274	^25^
PS93/025	025	80.481	−8.489	290.2	2.64	10213–536	^32,75^
PS100/270	270	79.497	−18.140	424	9.51	10190–1129	^74^
DA17-NG-ST14-171G	171G	74.090	−19.431	341.2	4.20	12179– −42	^28^
DA17-NG-ST01-019G	019G	79.634	−6.051	322.5	1.50	25520–142	^24^
DA17-NG-ST12-135G	135G	77.127	−10.676	500.9	2.70	22012–12	Unpublished

## Material and methods

### Study material

The marine sediment cores used in this study cover most of the Holocene and are retrieved from the Northeast Greenland shelf (Fig. 1, Table 1).

### Assignment of benthic foraminiferal traits

For the purpose of this study we used seven published marine sediment cores and one new core (DA17-NG-ST12-135G) (Table 1, Fig. 1). For each marine sediment core, the associated benthic foraminiferal species were assigned certain traits based on previous ecological studies published in research papers and databases (see Table 2 and Table S1 in Supplementary). The included traits were defined for the majority of the benthic foraminiferal species present in the sediment cores (see Table S1 in Supplementary). In this study, the assignment of oxygen tolerances and living mode was primarily based on an extensive literature review; however, when no information on certain species’ oxygen tolerances and living modes were available, test morphology was applied (see Table 1). Nevertheless, this approach is correct in 75% of the cases^76^. For every core and for every sample depth, the benthic foraminiferal species were converted to traits. When possible, each species was assigned one of all the trait variations listed in Table 2. Each trait was treated separately; the trait variations were converted to percentages and summed up to a 100 percent for each trait. A full list of all the benthic foraminiferal species found in the material analysed for this paper and their assigned traits are listed in Table S1 in the Supplementary information.

**Table 2:** Overview of traits assigned to the benthic foraminiferal species from the studied marine sediment cores.

Trait	Trait variation	References
Pores	Perforate, imperforate	Original descriptions of species
Living strategy/mode	Epifaunal, infaunal	Original descriptions of species and^13,36,39,40,42,81^
Substrate preferences	Mud, muddy sand, sand, hard substrate	^13,81^
Test material	Agglutinated, calcareous	Original descriptions of species
Oxygen tolerance	Oxic, suboxic, dysoxic	^12,36–39,51^
Food preferences	Fresh food, low food, old organic material	^12,13,40,42,44,81^

### Principal component analysis

A principal component analysis (PCA) was conducted in MatLab to statistically evaluate the traits that explain most of the variability in the datasets. For this, the percentage data for each trait was normalized to z-scores ${z}_{i}=\frac{{x}_{i}-\overline{x}}{s }$, where *s* is the standard deviation.

### Rate of change analysis

The *R-Ratepool* (version 0.6.1) R package was used to determine the rate-of-change in the benthic foraminiferal traits data^77^. The *R-Ratepool* is a powerful numerical tool to estimate rapid changes in chronological data sets with changing temporal resolution. All data sets are smoothed and binned into working units with a moving window before the rate-of-change calculations. The rate-of-change is then calculated between adjacent working units as a dissimilarity coefficient. Significant rate-of-change peak points are detected by using a “non-linear trend” GAM method. Separate calculations of rate-of-change was carried out for the benthic foraminiferal trait percentage data in each core, as well as for each trait (living strategy, test material, substrate, perforation, dissolved oxygen requirements and food requirements). For a full description of the rate-of-change calculations, the reader is referred to the Supplementary information.

## Results

### Benthic foraminiferal traits of cores

**Figure 2:**
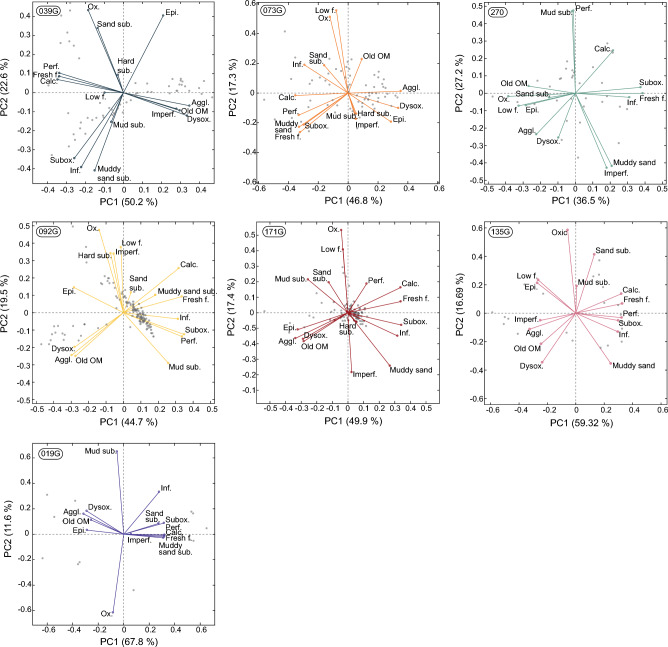
PCA biplots showing benthic foraminiferal trait loadings on PC1 and PC2 together with the explained variability. Grey dots are the transformed data for each sampling interval. *Perf.* = perforate, *Imperf.* = imperforate, *Epi.* = epifaunal, *Inf.* = infaunal, *sub.* = substrate, *Aggl.* = agglutinated, *Calc.* = calcareous, *Ox.* = oxic, *Subox.* = suboxic, *Dysox.* = dysoxic, *Fresh f.* = fresh food, *Low f.* = low food, *OM* = organic matter.

In the principal component analysis of the trait data (Fig. 2 and Table S2 in Supplementary), PC1 can generally be linked to foraminiferal test material as well as food preferences. The PC2 axis separates the benthic foraminiferal oxygen preferences. For cores 039G, 073G, 092G, 171G, and 135G, high positive and negative loadings on the PC2 axes correspond to oxic and dysoxic/suboxic tolerating benthic foraminifera, respectively (Fig. 2). In relation, the imperforate and perforate traits for core 270 show high negative and positive loadings on PC2 (Fig. 2); perforate benthic foraminifera are able to tolerate low dissolved oxygen content as opposed to imperforate species^45^.

**Figure 3:**
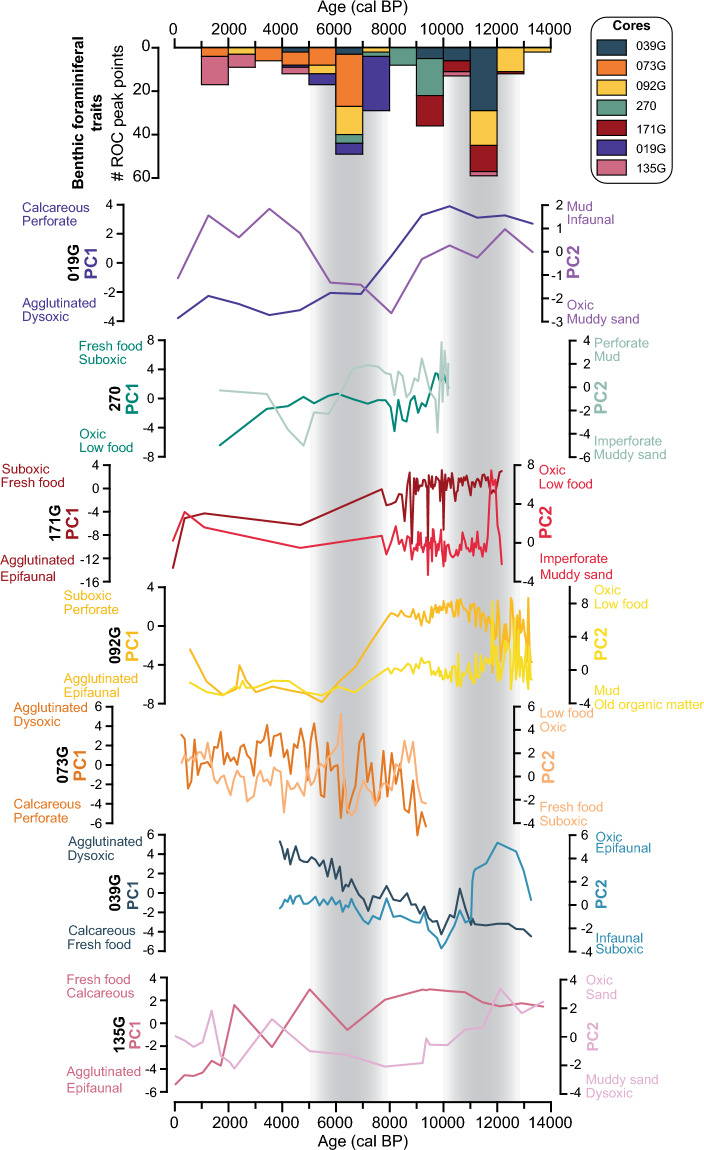
Summed up rate-of-change points for every 1000 years conducted on separate cores plotted with PC1 and PC2 time series for each core. The two traits with highest and lowest eigenvalues are listed on each principal component axis. Grey bars mark periods with the most substantial changes in the benthic foraminiferal trait data.

The highest scoring foraminiferal traits on the PC1 and PC2 time series show significant variability during the deglacial and Holocene period (ca. 12–11 cal ka BP) across all core sites (Fig. 3). This is also seen as a high rate-of-change identified in the peak point analysis of the separate traits (Fig. 3 and Fig. S1 in Supplementary): a large number of rate-of-change peak points is recorded in the traits of cores 092G, 039G, 171G, and 135G related to a transition in the overall abundance of oxic to dysoxic/suboxic tolerating benthic foraminiferal species. In comparison, species that prefer fresh food particles are abundant during the glacial (from 14 to 12 cal ka BP) at core sites 092G, 135G, 171G, 019G, and 039G (Fig. 3 and Fig. S1 in Supplementary).

From 10 to 9 cal ka BP, the traits in cores 039G, 270, and 171G show significant changes in the peak point detection analysis (Fig. 3 and Figures in Supplementary). Yet, the shifts do not occur within the same traits in the different cores. In core 039G, a decline in species preferring fresh food and a slight increase in species that feed on older organic matter is recorded during this time. Further south, the benthic foraminiferal fauna in core 270 shows a peak in fresh food and suboxic species alongside a sudden turnover from perforate to imperforate species at ca. 9.8 cal ka BP, followed by higher abundances of oxic species. The test material changes logged by the rate-of-change point analysis in core 171G are rapid and correspond to a few data points.

The traits in cores 039G, 073G, 092G, 270, and 019G show significant changes between 8 and 6 cal ka BP (Fig. 3 and Supplementary). Overall, a gradual turnover from a predominantly calcareous to an agglutinated fauna occur in the majority of the cores (019G, 092G, 073G, 039G) during this time. Relatively high number of rate-of-change peak points in the oxygen trait preferences in cores 019G and 073G point to significant changes and mark a transition towards dominance by dysoxia tolerant species. Adding to this, a gradual rise in dysoxic species is also observed at core sites 039G and 092G (Fig. 3 and Figs. S1, S2, S4 in Supplementary). Concurrently, cores 039G, 019G, and 073G exhibit a significant decline in species that prefer fresh food particles. Albeit the rate-of-change point analysis did not record significant changes in food preferences in core 092G, a decline in the fresh food preferring species is observed (Fig. S4 in Supplementary).

In the late Holocene (ca. 5 to 0 cal ka BP), shifts to a predominantly agglutinated, oxic and low food tolerant fauna are recorded in core 135G. Minor variability is also evident in cores 073G and 092G, where infaunal species become dominant between 3 and 2 cal ka BP in core 092G and between 2 and 1 cal ka BP in core 073G (Figs. S3 and S4 in Supplementary).

## Discussion

According to studies on benthic foraminiferal ecology, oxygen concentrations and food availability appear to be the two largest controlling factors on species distribution^40,53,78–80^. In fact, the link between benthic foraminiferal species distribution and water temperature and salinity is often too weak for detailed reconstructions of paleo environments (e.g.^53,81^). In the Arctic and North Atlantic region, similar conclusions have been made based on studies of both dead and stained benthic foraminiferal species, where different faunas were not directly linked to certain water temperatures or salinities, but rather to food supply ^78,82,83^. Dissolved oxygen is considered to control the absence and presence of certain species^53^, whereas increasing fluxes of organic matter cause proportional increases in abundance^14,53^. Especially opportunistic species benefitting from fresh food pulses can reproduce rapidly and thereby increase their abundance significantly^84^. Changes in the advection of the different water masses can also alter the bottom water oxygen content^85^. Additionally, the amount of dissolved oxygen at the sea floor is coupled with organic matter fluxes; high organic matter fluxes lead to low levels of oxygen at the sea floor due to organic matter degradation. Thus, distinguishing the effect of these two parameters can be difficult in the benthic foraminiferal traits.

While most benthic foraminiferal species have certain environmental requirements, not all are stenotopic. Many species are motile and can change their living position during times of stress. Some species tolerate a wide range of oxygen and food conditions but might have specific preferences under which they thrive. Nonetheless, the richness of species is highest in the top cm of the sea-floor sediments where higher food concentrations are available^53^. When oxygen becomes a limiting parameter at the sea floor, the epifaunal species are the first to disappear^86^. Under such circumstances, the burial efficiency of organic matter increases by 30%, beneficial for the true infaunal benthic foraminifera^87^.

Our results show that several changes in the benthic foraminiferal traits occurred across the shelf during the deglacial and Holocene period. Based on our PCA results, we suggest that overall, dissolved oxygen is the main abiotic environmental factor controlling the benthic foraminiferal faunal distribution during the deglacial and Holocene period. The second-most important factor seems to be food type preference.

### Response of benthic foraminiferal traits to environmental changes

#### The deglacial-Holocene transition (14–11 cal ka BP)

Throughout the end of the last glacial period (14–12 cal ka BP), benthic foraminiferal faunas that prefer oxic and low food conditions (cores 092G, 039G, 171G, 019G, and 135G) appear to dominate the shelf (Fig. 3). The disappearance of grounded ice and extensive sea-ice cover on the Northeast Greenland shelf might have caused low surface water productivity and sporadic fluxes of organic matter, causing low utilization of dissolved oxygen by aerobic organic matter degradation at the sea floor (cf.^53^). Erratic pulses of organic matter is utilized by species preferring fresh food^88,89^, as seen in cores 135G, 039G, 171G, and 019G.

**Figure 4:**
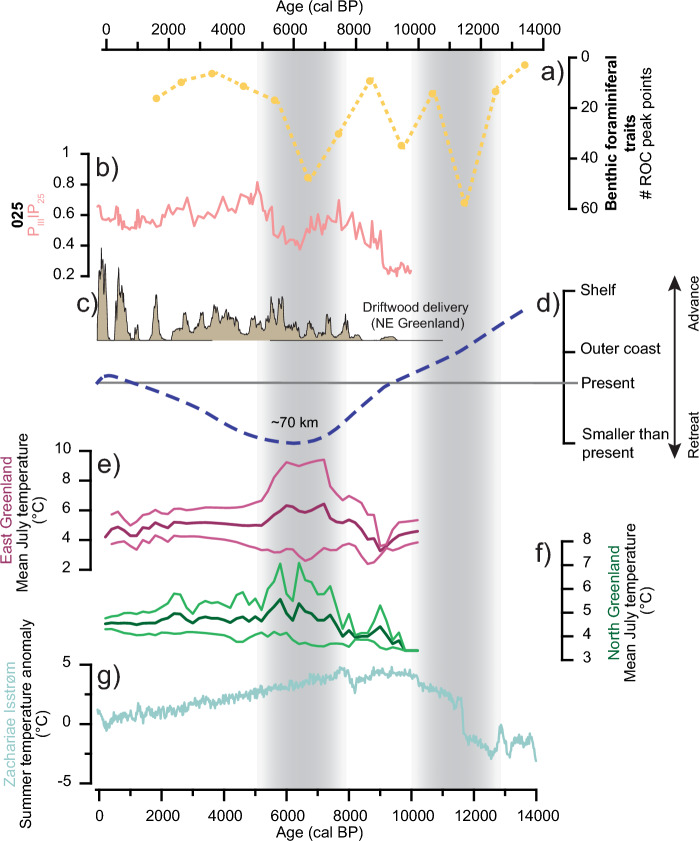
Comparison of benthic foraminiferal rate-of-change peak points and regional climate proxy data from Northeast Greenland. (**a**) The summed up number of benthic foraminiferal traits rate-of-change peak points, (**b**) the P_III_IP_25_ from core 025^32^, (**c**) driftwood delivery record from Northeast Greenland^31^, (**d**) the reconstructed NEGIS ice-margin fluctuations based on ^10^Be and ^14^C dates^30^, (**e,f**) reconstructed mean regional July temperatures (bold lines) with the regional 1 σ (thin lines) based on pollen records from East Greenland and North Greenland, respectively^29^, (**g**) modelled summer temperature anomaly at the ZI^90^.

The deglacial-Holocene transition (12–11 cal ka BP) marks rapid and significant changes in the traits of cores 092G, 039G, 171G, and 135G, where turnovers from oxic to dysoxic/suboxic species are observed. The sudden increase in air temperatures and landward retreat of the NEGIS (Fig. 4g, d)^26,30,90^ might have prompted increased primary production and thus a stronger food flux to the sea floor. The organic matter degradation at the sea floor caused lower oxygen content (cf.^53^), which dysoxic/suboxic species benefit from. Changes in ocean water mass flux may also have influenced the bottom-water oxygen content. Less influx of the relatively oxygen-rich Polar water component in exchange for a stronger Atlantic water advection might have caused reduced bottom water oxygen conditions. The investigations of the benthic foraminiferal species distributions of cores 092G, 039G, 171G, and 019G could primary decipher that the assemblages during the deglacial (14–12 cal ka BP) point to advection of relatively warmer Atlantic waters and cold surface water conditions^24, 26–28^. Thus, the interpretations based on the presence of the most dominant species during this time do not comment on the relatively high dissolved oxygen conditions as suggested by our trait interpretations. However, sporadic food pulses were detected at core sites 039G and 171G from 12 to 11 cal ka BP, related to the presence of specific indicator species^27,28^. Opposite, for core site 092G, the benthic foraminiferal assemblage analysis did not register the low food flux that we observed in the traits^26^ (Fig. 3 and Fig. S4 in Supplementary). The original benthic foraminiferal studies of cores 039G and 171G mention significant changes in the species distribution during the deglacial-Holocene transition (12–11 ka cal BP)^27,28^. However, in these assemblage studies the additional link to a change from oxic to dysoxic bottom-water conditions, as our trait data suggests, could not be seen. This demonstrates that further information can be extracted from the benthic foraminiferal trait data during this time.

#### Early-Mid Holocene warm period (11–6 cal ka BP)

Following the deglacial-Holocene transition, changes in the traits are controlled by regional environmental changes. From 10 to 9 cal ka BP several significant rate-of-change peak points are detected in cores 039G, 270 and 171G (Fig. 3). During this time, the Northeast Greenland shelf was subjected to high air temperatures, marginal sea-ice zone conditions and a NEGIS grounding zone located at the present day position (Fig. 4)^29,30,32,90^. These overall warm conditions affected the traits in distinct ways across the core sites. At core site 039G in the outer Westwind Trough, a shift from opportunistic fresh food benthic foraminifera to species able to utilize older organic matter is recorded (Fig. 3 and Fig. S2 in Supplementary). This is possibly related to the distant position and continuous landward retreat of the NEGIS (Fig. 4d), also indicated by lower sedimentation rates at core site 039G and a reduced sea-ice cover (Fig. 4b), fostering elevated surface primary production^27,30,32^. Enhanced surface water production, possibly related to changes in sea-ice configurations and/or changes in the advection of water masses increased the organic matter fluxes, which lead to increased consumption of oxygen at the sediment–water interface^45,83^. This could explain the relatively high numbers of infaunal benthic foraminiferal species utilizing degraded organic material^53,86^. Sporadic fluxes of food is recorded in the interpretations based on species distribution of core 039G related to a sea-ice environment with open water conditions^27^.

At the inner Northeast Greenland shelf, the laminated sediments in cores 270 and 092G reflect glacial proximal conditions with the presence of an ice tongue^26,74^. No significant changes are recorded in the traits of core 092G, despite core 270 showing several changes, potentially reflecting the very low sedimentation rates and low temporal resolution of core 092G. The changeover from a fresh food and suboxic dominated assemblage to a fauna with increasing numbers of imperforate oxic species might be a response to glacial and ice tongue proximity, prohibiting primary production and reduced sinking of organic particles, leading to well-oxygenated conditions at the sea floor^45,53^. A changeover to well-oxygenated bottom-waters is not recorded in the interpretations of the benthic species distributions during this time of core 270^74^.

In the southern-most core (171G), high biogenic silica concentrations points to an early Sirius Water Polynya formation and increased surface water production^28^. Based on this, the sporadic peaks in agglutinated species relate to corrosive brine formations prohibiting growth of calcareous tests^28,91^.

During the early part of the Mid Holocene (8–6 cal ka BP), widespread changes occurred in the traits across the shelf. The NEGIS was at its lowest Holocene extent, whilst high air temperatures prevailed in North and East Greenland inferred from pollen records coeval to reduced sea-ice conditions based on rare driftwood occurrences in North Greenland and a low P_III_IP_25_ (Fig. 4)^29–32^. The common significant changes in the traits across core sites relate to the shift in dominance of calcareous to agglutinated species in cores 019G, 092G, 073G, and 039G concurrently to a change towards a dysoxic tolerating fauna. The combination of increasing agglutinated individuals and dysoxia tolerating species together with low frequencies of fresh food preferring species might be linked to less fresh labile organic matter input from primary production. Agglutinated species are more opportunistic than calcareous species and can tolerate a lower food quality^13,91,92^. On the other hand, oxidation of organic matter can also lead to dissolution of calcareous tests and cause low dissolved oxygen levels at the sea floor^91,93,94^.

The interpretations based on benthic foraminiferal species distributions of cores 039G, 092G, 019G, and 073G relate the shift from calcareous to agglutinated species to a gradual decline in Atlantic water advection in favor of increased Polar water influence following overall warm bottom-water conditions^24–27^. The transition from fresh food to old organic matter preferring species is discussed in^26^ and^27^, based on changes in indicator species. However, the concurrent increasing numbers of dysoxic species is not noticed using the traditional assemblage analyses in any of the previous work^24–27^. Here we show that, while a change occurred in the dominant water mass, bottom-water oxygenation and food-availability played a significant role.

#### Mid to Late Holocene changes (6–0 cal ka BP)

The later part of the Holocene on the Northeast Greenland shelf is characterized by declining air temperatures, lower solar insolation, a readvance of the NEGIS, and an expansion of the sea-ice cover (Fig. 4). Exceptionally low primary production in conjunction with near perennial sea-ice conditions was followed by the final break-up of the 79NG ice shelf on the inner shelf at core site 270^74^. At core site 025 in the outer Westwind Trough, low ice rafted debris fluxes and increasing medium-sized silt percentages point to colder conditions and deposition of sea-ice sediments^75^. The fining of the sortable silt implies a weakening of the bottom currents, interpreted as lower advection of Atlantic-sourced water masses^75^. Contemporarily decreasing planktonic δ^18^O at core site 025 and increasing IP_25_ and HBI III values on the North Icelandic Shelf suggest a EGC strengthening and a south-eastward expansion of the Arctic front^95^.

Overall, only few significant rate-of change points are recorded in the traits. This is partially due to a lower temporal resolution in the datasets in many of the cores caused by lower sedimentation rates. Further, the benthic foraminiferal records for cores 039G and 270 end at 3.9 and 1.7 cal ka BP, respectively. The most extensive changes occur in core 135G at the outer Norske Trough (Figs. 1 and 3). Here, an agglutinated fauna is dominant together with oxic and low food tolerant species. Low phytoplankton productivity together with harsh sea-ice conditions might have impeded a strong organic matter flux to the sea floor resulting in oligotrophic conditions, while brine formation may have contributed to carbonate dissolution as also seen in the present-day foraminiferal assemblages^80^. However, more high-resolution benthic foraminiferal records from this time period are required in order to fully assess the effect of the NE Greenland environmental changes on the benthic foraminiferal traits. Additionally, the low temporal resolution complicate the direct comparisons between interpretations based on assemblages and traits for the late Holocene.

### Advantages of applying a trait-based approach and future perspectives

This is the first case study of applying a benthic foraminiferal trait-based approach to assess past benthic ecosystem alterations as a response to drastic global and regional climate changes in the Arctic. There are many advantages in replacing benthic foraminiferal species by their traits. Trait composition might contribute with more information about the overall state of benthic ecosystems, compared to examining benthic foraminiferal species distributions alone. The main advantages of a trait-based approach is that traits represent key aspects of the regional physical environment^96^. In our study, we showed that vital parameters (e.g., food availability and dissolved oxygen) for ecosystem functioning changed throughout the deglacial and Holocene period as a response to climate-driven factors (increasing air temperatures, retreating ice sheet and sea ice). For example, during the deglacial period, our trait analyses captured relatively high dissolved bottom-water oxygen conditions together with sporadic fluxes of organic matter across the NE Greenland shelf, which was not noticed in the assemblage studies^24–28,74^. Additionally, the deglacial-Holocene transition marked a changeover from oxic to suboxic conditions based on our trait reconstructions, where only few of the assemblage studies mention changes in food flux and none leave any comments on changes in oxygen conditions. Further, during the Mid Holocene period, our trait-based approach recorded periods where the oxygen conditions changed, which again was not mentioned in the assemblage studies. Therefore, trait-based analyses allows the reconstruction of past bottom water oxygen conditions, which is often not as clearly seen in assemblage analyses. Hence, trait-based analyses may provide different regional information that might otherwise be overlooked, when studying benthic foraminiferal assemblages in the traditional way in paleoceanographic studies.

Studies indicate that the responses in functional traits (observational traits that influence organismal and ecosystem processes and performance^33^) are observed earlier compared to at the taxonomic level, when impacted by environmental changes^97^. However, in order to use benthic foraminiferal traits in this context, a standardized trait terminology and methodology must be established. This will allow broad scale comparability between changes in certain benthic foraminiferal traits at targeted time periods. A robust catalogue of benthic foraminiferal traits allows for trait-based modelling to understand and predict ecosystem dynamics, based on well-defined relationships between traits and environmental factors.

## Summary and conclusions

In this study, we assigned traits to benthic foraminiferal species from marine sediment cores retrieved across the Northeast Greenland shelf covering the deglacial and Holocene period. Principal component analyses and rate-of-change calculations were applied to the trait datasets to resolve which traits explain most of the variability and to determine significant temporal changes in the traits. According to our results, bottom water oxygen, test material, and food preferences explain most of the variability in the datasets. Additionally, the rate-of-change results show that several significant changes occurred in the traits during the past ~ 14,000 years, linked to changes in external forcing mechanisms.

During the deglacial period, our results show that benthic foraminiferal species that prefer well-oxygenated bottom-waters and low food conditions dominated the shelf, potentially related to the disappearing of proximal grounded ice and perennial sea ice, causing sporadic fluxes of organic matter to the seafloor. A significant change is marked during the deglacial-Holocene transition across the shelf, where a changeover from high oxic to suboxic tolerating species is recorded in our trait analyses. This changeover was potentially caused by a combination of drastically increasing air temperatures and the continuous retreat of the Northeast Greenland Ice stream, promoting elevated primary production and high fluxes of organic material to the sea floor.

The Early Holocene warm period was characterized by high air temperatures, reduced sea-ice conditions and the NEGIS at its present-day position. During this time, the traits responded to different local environmental conditions across the shelf, related to differences in the proximity of the NEGIS ice tongue, polynya formation and sea-ice.

During the Middle Holocene, a shift from a calcareous to an agglutinated and dysoxic tolerating fauna occurred in several cores. This was linked to less input of fresh labile organic matter, which agglutinated foraminifera benefitted from. However, oxidation of organic matter can also lead to dissolution of calcareous tests and cause low dissolved oxygen conditions.

The Late Holocene marks a period were only few significant trait changes occurred. This is potentially related to the fewer data points in this period.

In general, the benthic foraminiferal assemblage-based results and interpretations on the same data set as analysed here do not comment on changes in dissolved oxygen conditions at the sea floor, and only few studies recorded changes in food supply. Thus, applying a trait-based approach might capture key changes in past bottom-water oxygen conditions that traditional benthic foraminiferal assemblage studies do not record. However, the trait-based method faces several weaknesses; not all traits can be assigned to all species due to the lack of knowledge on ecological preferences of certain benthic foraminiferal species. Most species have specific preferences under which they thrive, yet some species can change their traits in response to different environmental conditions.

This case study represents one of the first studies in the Arctic to apply a trait-based method to reconstruct deglacial and Holocene changes in benthic ecosystems as a response to changes in climate and environment. Our study underscores the value of using benthic foraminiferal traits to complement traditional species assemblage analyses in understanding the response of benthic ecosystems to environmental changes.

### Supplementary Information

Supplementary Information.

## Data Availability

All foraminiferal data from the marine sediment cores are available from the PANGAEA database or as supporting information in the original publications except for the foraminiferal data of core DA17-NG-ST12-135G, which is unpublished.
