# Supplementary material for: Response of Arctic benthic foraminiferal traits to past environmental changes
Source: Sci Rep. 2023 Dec 13;13:22135. doi: 10.1038/s41598-023-47603-w (PMC10719382; doi:10.1038/s41598-023-47603-w)
Supplement: Supplementary file 1 — Supplementary Information. [file 41598_2023_47603_MOESM1_ESM.pdf]

Supplementary: Response of Arctic benthic foraminiferal traits to past environmental changes

Katrine Elnegaard Hansen[1, 2*], *Christof Pearce*[1], *Marit-Solveig Seidenkrantz*[1]

[1]Paleoceanography and Paleoclimate Group, Department of Geoscience, Arctic Research Centre, and iClimate, Aarhus University, Aarhus, Denmark

[2]The geological survey of Denmark and Greenland (GEUS), Department of Near Surface Land and Marine Geology, Aarhus, Denmark

Correspondence to: Katrine Elnegaard Hansen (katrine.elnegaard@geo.au.dk)

Methods

Rate of change analysis

The computation of rate-of-change for all dataset types is executed in multiple steps; 1) data smoothing and creation of time bins, 2) sub-setting levels/sample depths in each time bin into working units, 3) calculation of rate-of-change scores between working units. These steps were repeated and randomized 10,000 times and all the rate-of-change score results are summarized. Following the calculations of rate-of-change in the datasets, significant and rapid changes in the datasets were evaluated by peak-point detection. The peak-point detection was performed by applying a "Non-linear trend" method, which is a conservative generalized additive model (GAM) that is fitted through the rate-of-change scores and associated ages, using the mgcv R package[1]. The residual is then calculated as the distance between each point and the fitted value, and the standard deviation is calculated for all residuals. If a peak is 1.5 standard deviations higher than the fitted model, it is considered a point of significant change.

The separate percentage trait data was smoothed by using the built in age-weighted average smoothing method over 5 points and time bins of 500 years were created, except for core 019G and 135G, where the bin sizes were increased to 1500 and 1000, respectively, due to the low temporal resolution of the foraminiferal data. The rate-of-change for each set of trait data was calculated between consecutive working units, as a dissimilarity coefficient. The working units were defined using binning with a moving window of 500 years with five window shifts, resulting in a rate of change estimation every 100 years of the core, except for cores 135G and 019G, where the bin sizes were increased; here the rate of change was estimated for every 200 and 300 years,

respectively[2]. The binning was repeated 5 times and the results from all window positions were combined into one single value. Defining the working units with the "binning with a moving window" method makes up for changing sedimentation rates and shifting resolution of the data in the defined time bins[2]. The Chi-squared coefficient was used to calculate the dissimilarity i.e. the rate-of-change between the working units, as recommended by Mottl et al. (2021b) for closed compositional data (percentages). Detection of significant changes (peak points) in traits over time were estimated by using the "Non-linear trend" method.

Table S1: Benthic foraminiferal species and their assigned traits.

**Species**	**Living strategy**	**Test material**	**Substrate preferences**	**Pores**	**Oxygen tolerance**	**Food preferences**
Adercotryma glomeratum	Epifaunal	Agglutinated	Muddy sand	No	Dysoxic	Fresh food
Alabaminella weddellensis	Epifaunal	Calcareous		Yes	Oxic	Fresh food
Ammodiscus sp.	Epifaunal	Agglutinated	Mud	No	Dysoxic	Old organic matter
Ammoglobigerina globigeriniformis	Infaunal	Agglutinated	Mud			
Ammonia sp.	Infaunal	Calcareous	Muddy	Yes	Dysoxic	Old organic material
Ammotium cassis	Infaunal	Agglutinated	Mud	No	Dysoxic	Old organic material
Astrononion gallowayi	Epifaunal	Calcareous	Mud	Yes	Suboxic	Old organic material
Bolivina albatrossi	Infaunal	Calcareous	Mud	Yes	Dysoxic	
Bolivina arctica	Infaunal	Calcareous	Mud	Yes	Dysoxic	Old organic material
Bolivina pseudopunctata	Infaunal	Calcareous	Mud	Yes	Dysoxic	Fresh food
Bolivina sp.	Infaunal	Calcareous			Dysoxic	
Bolivina variabilis	Infaunal	Calcareous		Yes	Dysoxic	Fresh food
Buccella frigida	Epifaunal	Calcareous	Muddy sand	Yes	Oxic	

Buccella hannai subsp. arctica	Epifaunal	Calcareous	Mud	Yes		
Buccella sp.	Epifaunal	Calcareous				
Buccella tenerrima	Epifaunal	Calcareous				
Buliminella elegantissima	Infaunal	Calcareous	Muddy sand	Yes	Dysoxic	Old organic material
Cassidulina neoteretis	Infaunal	Calcareous	Mud	Yes	Suboxic	Fresh food
Cassidulina reniforme	Infaunal	Calcareous	Muddy sand	No	Suboxic	Fresh food
Ceratobulimina arctica	Infaunal	Calcareous	Mud	Yes	Suboxic	
Cibicides lobatulus	Epifaunal	Calcareous	Sand	Yes	Oxic	Fresh food
Cibicides lobatulus/Cibicides fletcheri	Epifaunal	Calcareous	Sand	Yes	Oxic	Fresh food
Cibicidoides globulosus	Epifaunal	Calcareous		Yes	Oxic	Fresh food
Cibicidoides wuellerstorfi	Epifaunal	Calcareous	Sand	Yes	Oxic	Low food, fresh food
Cornuspira involvens	Epifaunal	Calcareous		No		
Cribrostomoides conglobatus		Agglutinated				
Cribrostomoides crassimargo	Epifaunal	Agglutinated	Sand	No		Old organic material
Cribrostomoides kosterensis	Epifaunal	Agglutinated				
Cribrostomoides sp.	Epifaunal	Agglutinated		No		
Cribrostomoides subglobosa	Epifaunal	Agglutinated	Mud	No	Oxic	Low food, fresh food

Cribtostomoides jeffreysii	Epifaunal	Agglutinated	Sand	No		Old organic material
Cuneata arctica	Infaunal	Agglutinated	Muddy sand	No		Old organic material
Cyclogyra distincta		Calcareous		No		
Cyclogyra sp.	Epifaunal	Calcareous	Sandy	No		
Dentalina sp.	Infaunal	Calcareous	Mud		Suboxic	Low food
Deuterammina grahami	Epifaunal	Agglutinated				
Deuterammina montagui	Epifaunal	Agglutinated				
Deuterammina sp.	Epifaunal	Agglutinated				
Earlandammina inconspicua	Epifaunal	Agglutinated	Muddy sand			
Eggerelloides advenus	Infaunal	Agglutinated	Muddy sand	No	Dysoxic	Old organic material
Elongobula sp.		Calcareous		Yes		
Elphidium albiumbilicatum	Infaunal	Calcareous	Muddy sand	Yes	Dysoxic	Old organic material
Elphidium asklundi	Epifaunal	Calcareous	Muddy sand	Yes		
Elphidium clavatum	Infaunal	Calcareous	Muddy sand	Yes	Suboxic	Fresh food
Elphidium frigidum	Infaunal	Calcareous	Mud	Yes		Old organic material
Elphidium hallandense	Epifaunal	Calcareous	Silty sand	Yes	Dysoxic	
Elphidium sp.	Epifaunal	Calcareous		Yes		
Eoeponidella pulchella	Epifaunal	Calcareous	Sand	Yes	Oxic	
Epistominella arctica	Epifaunal	Calcareous	silty sandy clay	Yes	Oxic	Fresh food
Epistominella sp.	Epifaunal	Calcareous			Oxic	Fresh food

Epistominella takayanagii	Epifaunal	Calcareous	Clay			Fresh food
Epistominella vitrea	Infaunal	Calcareous	Mud	Yes	Oxic	Fresh food
Favulina hexagona	Infaunal	Calcareous	Mud		Suboxic	
Favulina melo	Infaunal	Calcareous	Mud		Suboxic	
Favulina squamosa	Infaunal	Calcareous	Mud		Suboxic	
Fissurina laevigata	Infaunal	Calcareous		No	Suboxic	
Fissurina orbignyana	Infaunal	Calcareous	Muddy sand	Yes	Suboxic	
Fissurina sp.	Infaunal	Calcareous				
Florilus sp.	Infaunal	Calcareous	Muddy	Yes	Suboxic	
Fursenkoina sp.	Infaunal	Calcareous	Muddy sand	Yes	Dysoxic	Old organic material
Gavelinopsis praegeri	Epifaunal	Calcareous	Hard substrate	Yes	Oxic	Fresh food
Glabratella arctica	Epifaunal	Calcareous	Sandy, hard substrate	Yes	Oxic	Fresh food
Globobulimina auriculata subsp. arctica	Infaunal	Calcareous	Mud	Yes	Dysoxic	
Globobulimina sp.	Infaunal	Calcareous	Mud	Yes	Dysoxic	Old organic material
Globocassidulina subglobosa	Infaunal	Calcareous	Mud	Yes	Oxic	Fresh food
Globulina gibba	Infaunal	Calcareous		Yes		
Glomospira charoides	Epifaunal	Agglutinated			Suboxic	
Glomospira sp.	Epifaunal	Agglutinated		No	Dysoxic	Old organic matter
Glomulina oculus	Epifaunal	Calcareous	Sand	No	Oxic	
Guttulina glacialis	Epifaunal	Calcareous		Yes		
Guttulina sp.	Infaunal	Calcareous		Yes		

Gyroidina lamarckiana	Epifaunal	Calcareous	Mud	Yes	Suboxic	
Gyroidina neosoldanii	Epifaunal	Calcareous	Mud	Yes	Suboxic	
Gyroidina orbicularis	Epifaunal	Calcareous	Mud	Yes	Suboxic	
Gyroidina sp.	Epifaunal	Calcareous	Mud	Yes	Suboxic	
Hansenisca soldanii	Epifaunal	Calcareous	Mud		Suboxic	
Haplophragmoides sp.						
Haynesina nivea	Infaunal	Calcareous	Mud, silt	Yes		Fresh food
Haynesina orbiculare	Infaunal	Calcareous	Muddy sand	Yes	Suboxic	
Hormosinella guttifera	Infaunal	Agglutinated		No	Suboxic	Old organic matter, low food
Hormosinella sp.	Infaunal	Agglutinated	Mud	No		
Hyperammina sp.		Agglutinated				Fresh food
Islandiella helenae	Infaunal	Calcareous	Sand	Yes		Fresh food
Islandiella norcrossi	Infaunal	Calcareous	Muddy sand	Yes		Fresh food
Islandiella sp.	Infaunal	Calcareous	Muddy sand	Yes		Fresh food
Laevidentalina elegans	Infaunal	Calcareous	Mud		Suboxic	Low food
Lagena distoma	Infaunal	Calcareous	Muddy sand		Suboxic	
Lagena elongata	Infaunal	Calcareous	Clay		Suboxic	
Lagena laevis	Infaunal	Calcareous			Suboxic	
Lagena mollis	Infaunal	Calcareous	Mud		Suboxic	
Lagena semilineata	Infaunal	Calcareous			Suboxic	
Lagena sp.	Infaunal	Calcareous			Suboxic	
Lagena striata	Infaunal	Calcareous			Suboxic	

Lagena sulcata var. laevicostata	Infaunal	Calcareous			Suboxic	
Lagenammina difflugiformis	Infaunal	Agglutinated	Sandy		Dysoxic	Old organic material
Lagenammina tubulata	Infaunal	Agglutinated	Sandy		Dysoxic	Old organic material
Lenticulina sp.						
Leptohalysis catella	Infaunal	Agglutinated	Muddy sand	No	Dysoxic	Fresh food
Lobatula lobatula	Epifaunal	Calcareous	Sand, hard substrate	Yes		Low food
Melonis barleeanus	Infaunal	Calcareous	Mud	Yes	Suboxic	Old organic material
Miliolida sp.	Epifaunal	Calcareous		No	Oxic	
Miliolinella chukchiensis	Epifaunal	Calcareous		No	Oxic	Fresh food
Miliolinella subrotunda	Epifaunal	Calcareous	Hard substrate	No	Oxic	Fresh food
Nodosaria sp.						
Nonionella auricula	Infaunal	Calcareous	Mud	Yes	Suboxic	
Nonionella fragilis	Infaunal	Calcareous	Mud		Suboxic	
Nonionella iridea	Infaunal	Calcareous	Mud	Yes	Suboxic	Old organic material
Nonionella sp.	Infaunal	Calcareous	Mud	Yes	Suboxic	
Nonionellina labradorica	Infaunal	Calcareous	Muddy sand	Yes	Suboxic	Fresh food
Nonionoides turgidus	Infaunal	Calcareous	Mud	Yes	Suboxic	Fresh food
Oolina borealis	Infaunal	Calcareous	Mud		Suboxic	
Oolina williamsoni	Infaunal	Calcareous	Mud		Suboxic	
Oridorsalis tenerus	Epifaunal	Calcareous		Yes	Suboxic	

Oridorsalis umbonatus	Epifaunal	Calcareous	Mud	Yes	Oxic	Low food
Parafissurina arctica	Infaunal	Calcareous	Mud	Yes	Suboxic	
Parafissurina fusiformis	Infaunal	Calcareous	Mud		Suboxic	
Parafissurina himatiostoma	Infaunal	Calcareous		Yes		Fresh food
Parafissurina lateralis	Infaunal	Calcareous		Yes		
Parafissurina sp.	Infaunal	Calcareous		Yes		
Paratrochammina challengeri	Epifaunal	Agglutinated	Mud		Oxic	Low food
Patellina corrugata	Epifaunal	Calcareous	Hard substrate	Yes	Oxic	Fresh food
Polymorphina sp.		Calcareous				
Portatrochammina antarctica subsp. antarctica	Epifaunal	Agglutinated	Mud			Fresh food
Portatrochammina bipolaris	Epifaunal	Agglutinated	Silty clay		Dysoxic	
Psammophera sp.		Agglutinated				
Psammosphaera fusca	Epifaunal	Agglutinated	Sand, clay		Dysoxic	Old organic material
Pseudopolymorphina soldanii	Infaunal	Calcareous		Yes		
Pullenia bulloides	Infaunal	Calcareous	Mud		Suboxic	Fresh food
Pullenia osloensis	Infaunal	Calcareous	Clay	Yes	Suboxic	
Pullenia quinqueloba	Infaunal	Calcareous	Mud	Yes	Suboxic	Old organic matter
Pullenia sp.	Infaunal	Calcareous	Mud	Yes	Suboxic	Old organic matter

Pullenia subcarinata	Infaunal	Calcareous	Mud	Yes	Suboxic	Old organic matter
Pyrgo williamsoni	Epifaunal	Calcareous	Hard substrate	No	Oxic	Fresh food
Quinqueloculina akneriana	Epifaunal	Calcareous		No	Oxic	Fresh food
Quinqueloculina arctica	Epifaunal	Calcareous		No	Oxic	
Quinqueloculina lamarckiana	Epifaunal	Calcareous	Muddy sand	No	Oxic	
Quinqueloculina seminulum	Epifaunal	Calcareous	Sandy clay	No	Oxic	Fresh food
Quinqueloculina sp.	Epifaunal	Calcareous		No	Oxic	Fresh food
Quinqueloculina stalkeri	Epifaunal	Calcareous	Mud	No	Oxic	Fresh food
Recurvoides contortus	Epifaunal	Agglutinated	Silt	No		Old organic material
Recurvoides sp.	Epifaunal	Agglutinated				
Recurvoides trochamminiforme	Epifaunal	Agglutinated		No	Oxic	Old organic material
Recurvoides turbinatus	Epifaunal	Agglutinated	Mud	No	Oxic	Old organic material
Reophax bilocularis	Epifaunal	Agglutinated				Fresh food
Reophax catenatus	Infaunal	Agglutinated		No		Old organic matter
Reophax fusiformis	Infaunal	Agglutinated	Muddy sand	No	Oxic	Low food, fresh food
Reophax pilulifer	Infaunal	Agglutinated	Sandy	No		Fresh food
Reophax scorpiurus	Epifaunal	Agglutinated	Sandy	No		Fresh food
Reophax sp.	Infaunal	Agglutinated	Muddy sand	No	Oxic	

Reophax subfusiformis	Infaunal	Agglutinated	Muddy sand	No	Oxic	Low food
Reophax subfusiformis/gaussicus/pilulifera	Infaunal	Agglutinated	Muddy sand	No		
Rhabdammina sp.						
Robertalinoides						
Robertina arctica	Infaunal	Calcareous	Sandy clay	Yes		
Saccammina sphaerica	Infaunal	Agglutinated	Sand			Old organic matter
Sagrina sp.	Infaunal	Calcareous			Suboxic	
Silicosigmoilina groenlandica	Epifaunal	Calcareous		No	Oxic	Fresh food
Spirillina sp.						
Spiroplectammina biformis	Infaunal	Agglutinated	Muddy sand	No	Dysoxic	
Stainforthia concava	Infaunal	Calcareous	Muddy sand	Yes	Dysoxic	Fresh food
Stainforthia feylingi	Infaunal	Calcareous	Muddy sand	Yes	Dysoxic	Fresh food
Stainforthia fusiformis	Infaunal	Calcareous	Muddy sand	Yes	Dysoxic	Fresh food
Stainforthia loeblichi	Infaunal	Calcareous	Muddy sand	Yes	Dysoxic	Fresh food
Stainforthia sp.	Infaunal	Calcareous	Muddy sand	Yes	Dysoxic	Fresh food
Stetsonia horvathi	Infaunal	Calcareous	Silty sandy clay		Oxic	Low food
Textularia earlandi	Infaunal	Agglutinated	Silty clay	No	Dysoxic	Low food
Textularia kattegatensis	Infaunal	Agglutinated	Mud	No	Suboxic	Fresh food
Textularia sp.	Infaunal	Agglutinated				
Textularia torquata	Infaunal	Agglutinated	Mud	No		
Trifarina fluens	Infaunal	Calcareous	Muddy sand		Oxic	Fresh food

Triloculina sp.	Epifaunal	Calcareous	Mud, sand	No	Oxic	Fresh food
Triloculina tricarinata	Epifaunal	Calcareous	Sandy clay	No	Oxic	
Triloculina trihedra	Epifaunal	Calcareous	Sandy clay	No	Oxic	
Trochammina nana	Epifaunal	Agglutinated				
Trochammina ochracea	Epifaunal	Agglutinated	Sand	No		
Trochammina sp.	Epifaunal	Agglutinated		No		
Valvulineria arctica/Nonionella fragilis	Infaunal	Calcareous	Mud	Yes	Suboxic	

Table S2: PCA eigenvalues for each trait in each core.

**039G**			**073G**			**092G**		
Trait	PC1	PC2	Trait	PC1	PC2	Trait	PC1	PC2
Agglutinated	0.35	-0.07	Agglutinated	0.35	0.01	Agglutinated	-0.30	-0.24
Calcareous	-0.35	0.07	Calcareous	-0.35	-0.02	Calcareous	0.32	0.26
Dysoxic	0.34	-0.12	Dysoxic	0.34	-0.10	Dysoxic	-0.28	-0.20
Epifaunal	0.21	0.41	Epifaunal	0.29	-0.19	Epifaunal	-0.28	0.14
Fresh food	-0.34	0.09	Fresh food	-0.32	-0.26	Fresh food	0.34	0.09
Hard substrate	-0.03	0.09	Hard substrate	0.05	-0.14	Hard substrate	-0.07	0.34
Imperforate	0.28	-0.08	Imperforate	0.05	-0.17	Imperforate	-0.06	0.34
Infaunal	-0.22	-0.39	Infaunal	-0.29	0.19	Infaunal	0.31	-0.04
Low food	-0.10	0.00	Low food	-0.08	0.56	Low food	-0.01	0.38
Mud substrate	-0.06	-0.15	Mud substrate	0.06	-0.14	Mud substrate	0.26	-0.29
Muddy sand substrate	-0.15	-0.41	Muddy sand substrate	-0.30	-0.21	Muddy sand substrate	0.18	0.10
Old organic material	0.30	-0.09	Old organic material	0.09	0.23	Old organic material	-0.27	-0.25
Oxic	-0.19	0.43	Oxic	-0.12	0.51	Oxic	-0.14	0.47
Perforate	-0.34	0.10	Perforate	-0.33	-0.15	Perforate	0.34	-0.14

Sand substrate	-0.14	0.34	Sand substrate	-0.16	0.19	Sand substrate	0.04	0.12
Suboxic	-0.26	-0.34	Suboxic	-0.33	-0.23	Suboxic	0.35	-0.13

**270**			**019G**			**135**		
Trait	PC1	PC2	Trait	PC1	PC2	Trait	PC1	PC2
Agglutinated	-0.22	-0.24	Agglutinated	-0.31	0.16	Agglutinated	-0.32	-0.11
Calcareous	0.22	0.25	Calcareous	0.32	-0.01	Calcareous	0.32	0.14
Dysoxic	-0.10	-0.26	Dysoxic	-0.29	0.18	Dysoxic	-0.24	-0.35
Epifaunal	-0.28	-0.07	Epifaunal	-0.29	0.03	Epifaunal	-0.27	0.21
Fresh food	0.39	0.00	Fresh food	0.32	-0.02	Fresh food	0.33	0.07
Imperforate	0.18	-0.43	Imperforate	0.06	0.00	Imperforate	-0.25	-0.05
Infaunal	0.31	-0.02	Infaunal	0.28	0.33	Infaunal	0.30	-0.13
Low food	-0.33	-0.07	Mud substrate	-0.05	0.65	Low food	-0.26	0.23
Mud substrate	-0.02	0.47	Muddy sand substrate	0.31	-0.03	Mud substrate	0.01	0.19
Muddy sand substrate	0.21	-0.42	Old organic material	-0.26	0.11	Muddy sand substrate	0.25	-0.35
Old organic material	-0.27	0.04	Oxic	-0.08	-0.62	Old organic material	-0.24	-0.22
Oxic	-0.38	-0.02	Perforate	0.32	0.00	Oxic	-0.06	0.58
Perforate	-0.01	0.47	Sand substrate	0.27	0.09	Perforate	0.32	-0.03
Sand substrate	-0.15	-0.02	Suboxic	0.32	0.09	Sand substrate	0.13	0.41
Suboxic	0.38	0.03				Suboxic	0.30	-0.05

**171G**		
Trait	PC1	PC2
Agglutinated	-0.34	-0.17
Calcareous	0.34	0.16
Dysoxic	-0.29	-0.17
Epifaunal	-0.32	-0.11
Fresh food	0.34	0.07
Hard substrate	0.05	-0.06
Imperforate	0.02	-0.38

Infaunal	0.32	-0.15
Low food	-0.03	0.41
Mud substrate	-0.26	0.21
Muddy sand substrate	0.27	-0.34
Old organic material	-0.28	-0.18
Oxic	-0.04	0.53
Perforate	0.12	0.19
Sand substrate	-0.12	0.20
Suboxic	0.34	-0.08

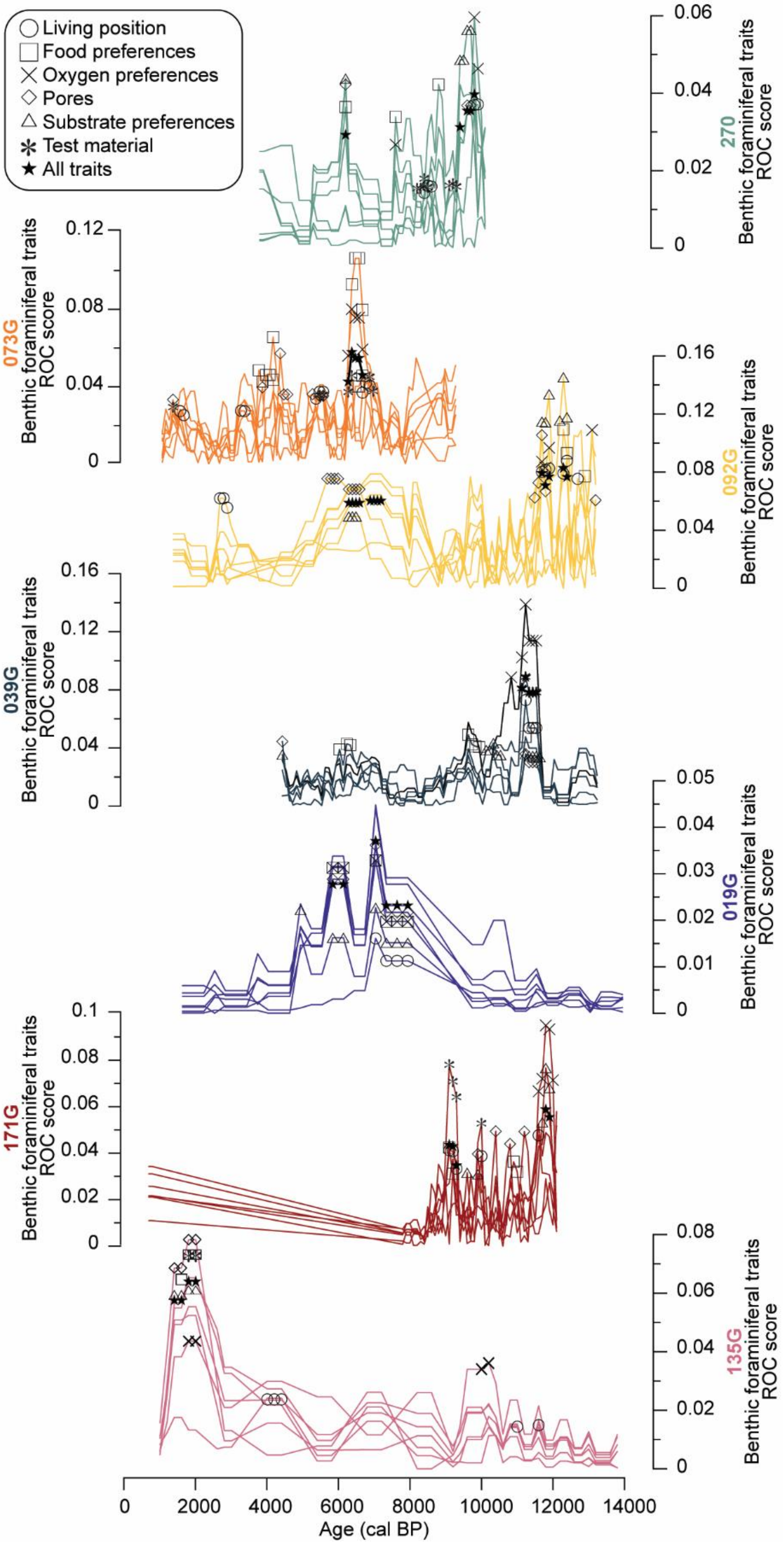

Living position
Food preferences
Oxygen preferences
Pores
Substrate preferences
Test material
All traits
270
Benthic foraminiferal traits ROC score
073G
Benthic foraminiferal traits ROC score
092G
Benthic foraminiferal traits ROC score
039G
Benthic foraminiferal traits ROC score
019G
Benthic foraminiferal traits ROC score
171G
Benthic foraminiferal traits ROC score
135G
Benthic foraminiferal traits ROC score
Age (cal BP)
0
2000
4000
6000
8000
10000
12000
14000

Figure S1: Benthic foraminiferal rate-of-change scores for each trait and core. The peak point detection results indicate significant changes and are marked by symbols (see legend).

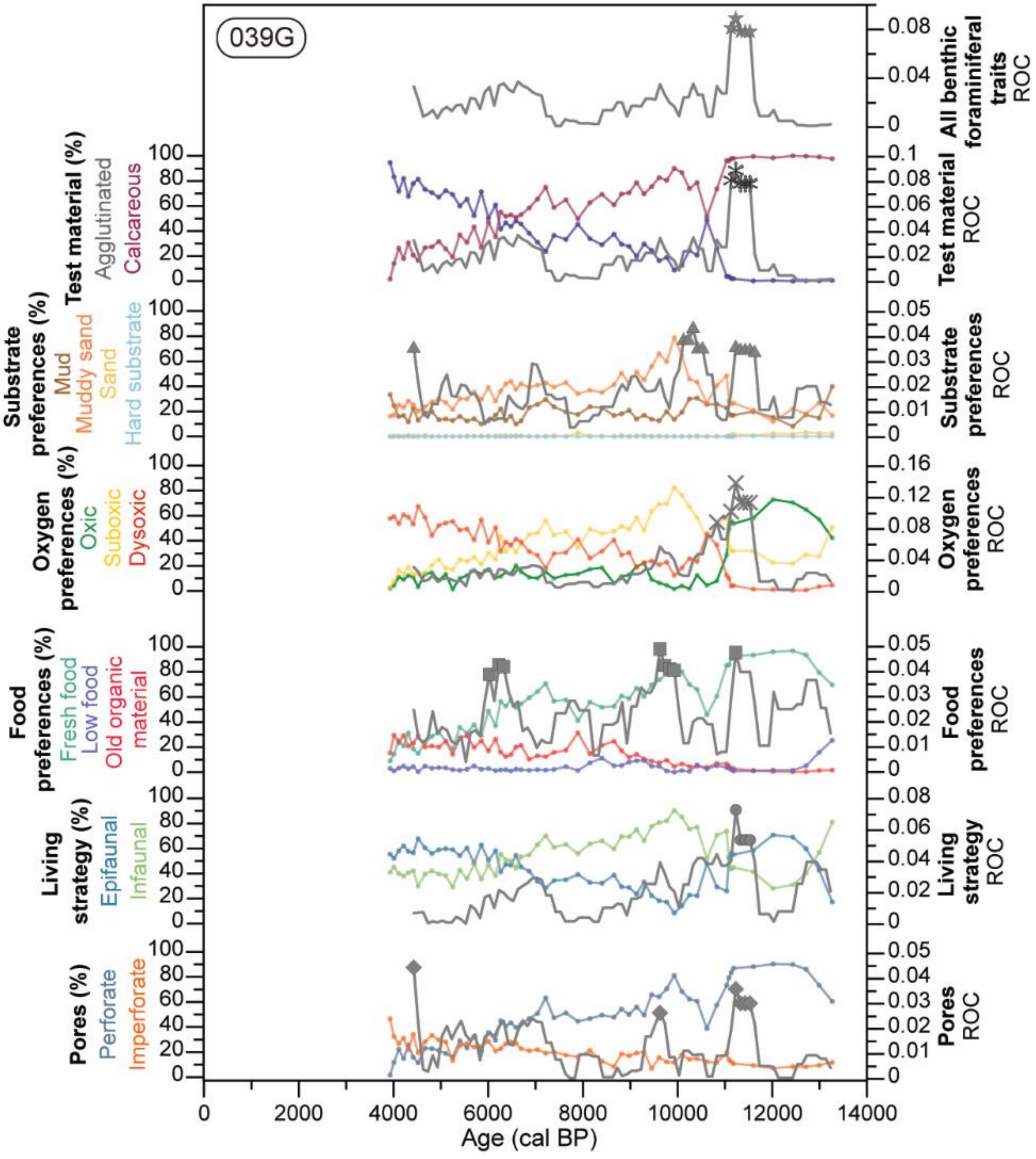

Figure S2: Changes in the benthic foraminiferal traits over time (%) and separate rate-of-change estimates together with significant peak points for each trait for core 039G.

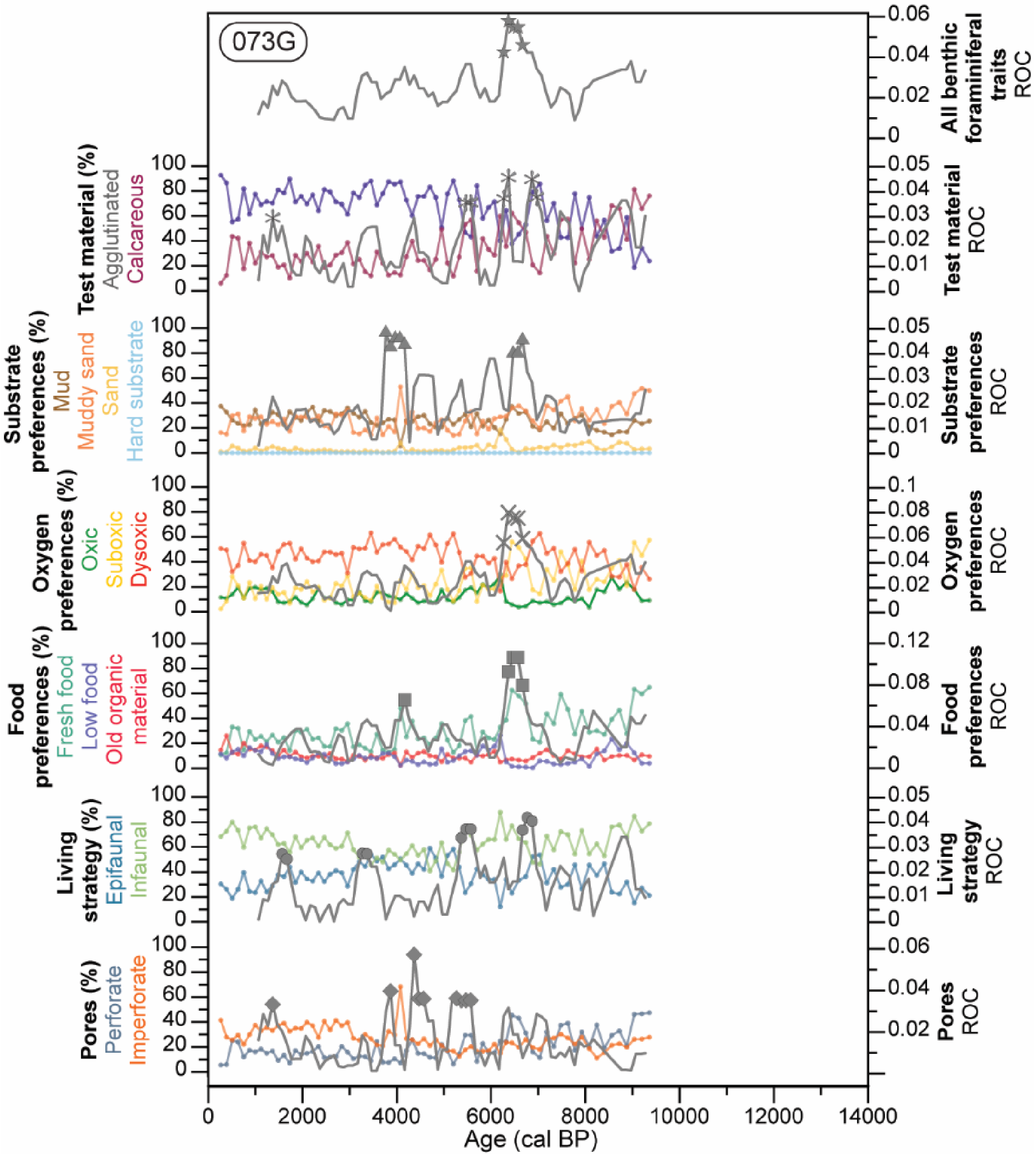

Figure S3: Changes in the benthic foraminiferal traits over time (%) and separate rate-of-change estimates together with significant peak points for each trait for core 073G.

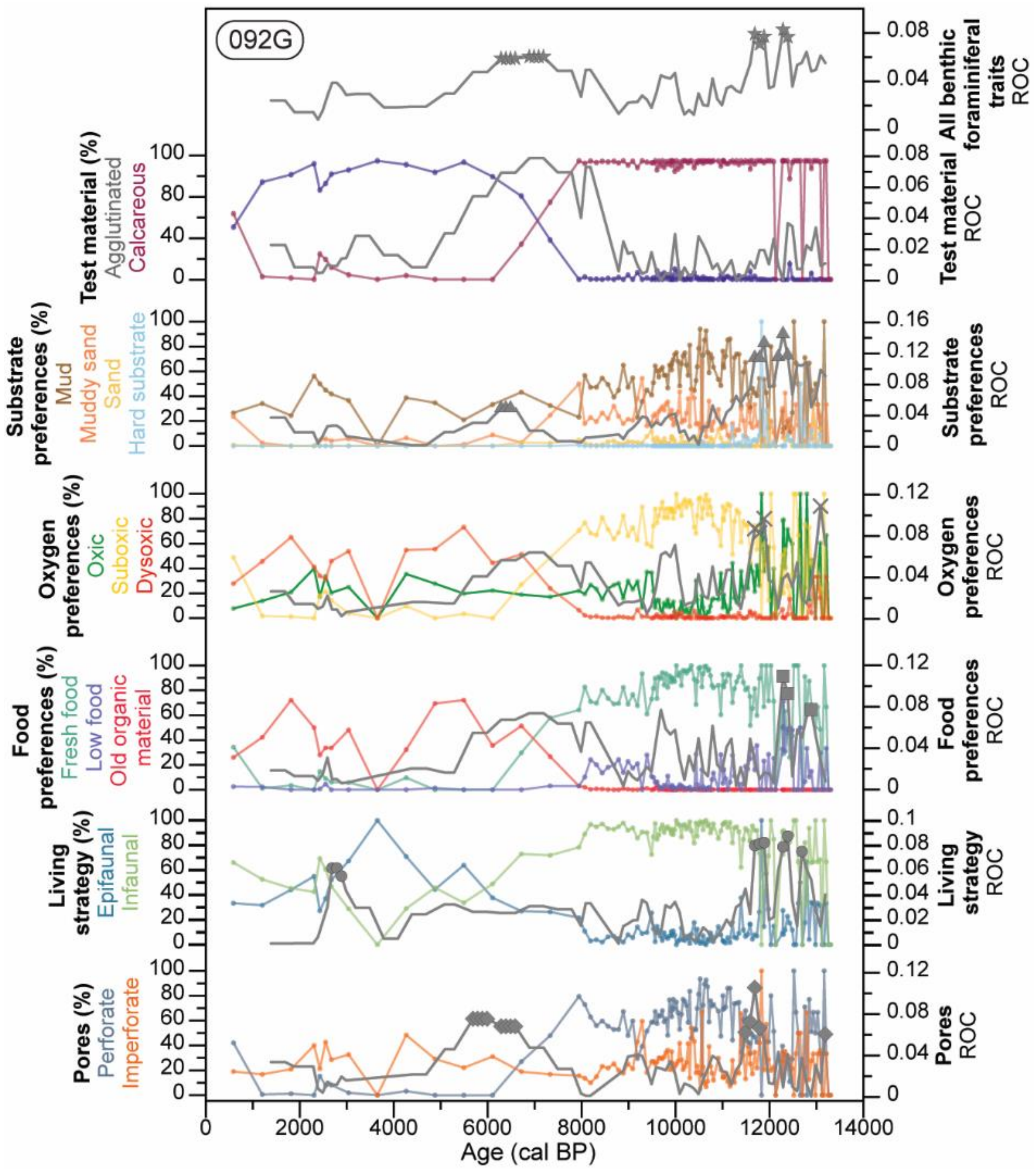

Figure S4: Changes in the benthic foraminiferal traits over time (%) and separate rate-of-change estimates together with significant peak points for each trait for core 092G.

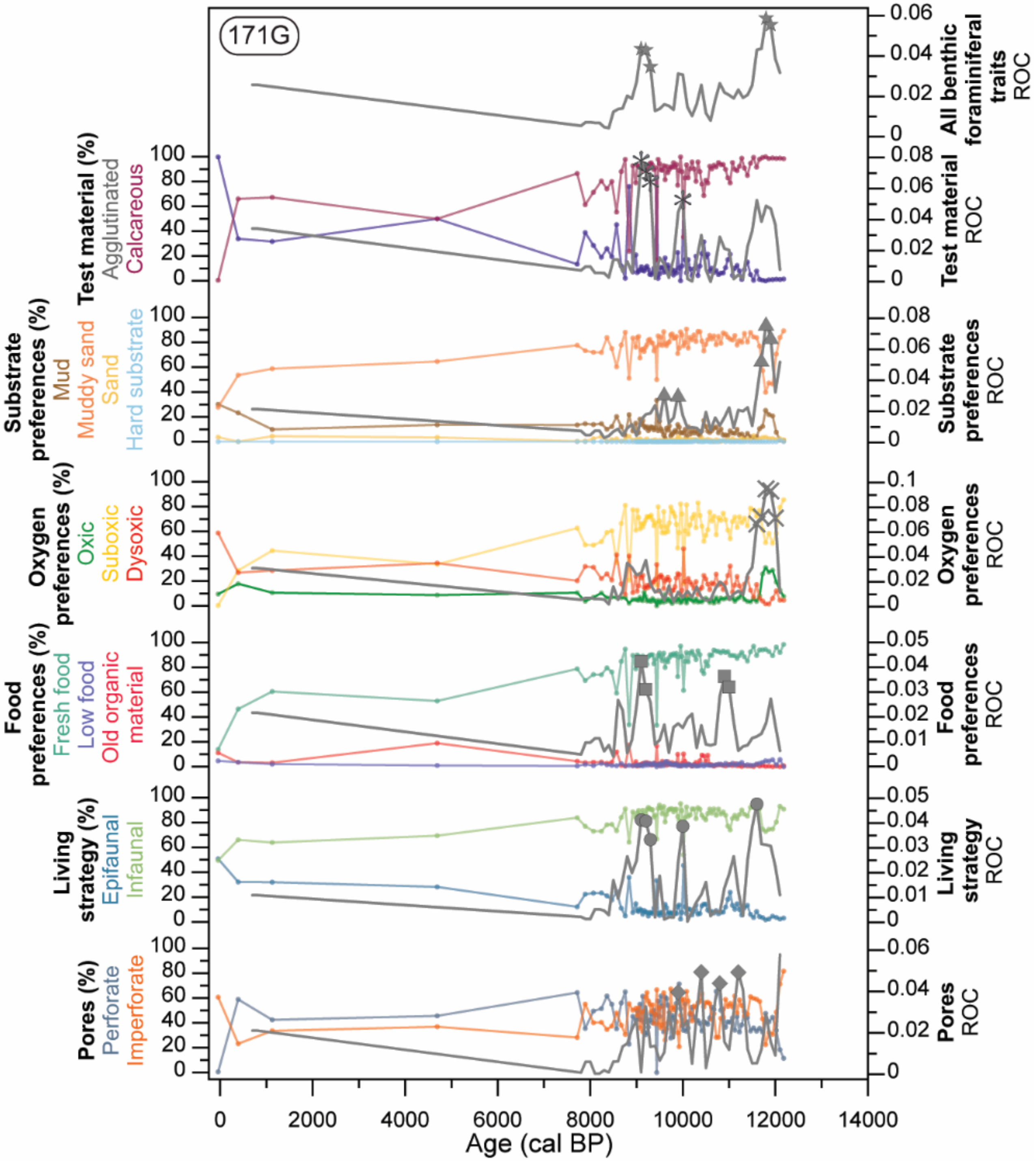

Figure S5: Changes in the benthic foraminiferal traits over time (%) and separate rate-of-change estimates together with significant peak points for each trait for core 171G.

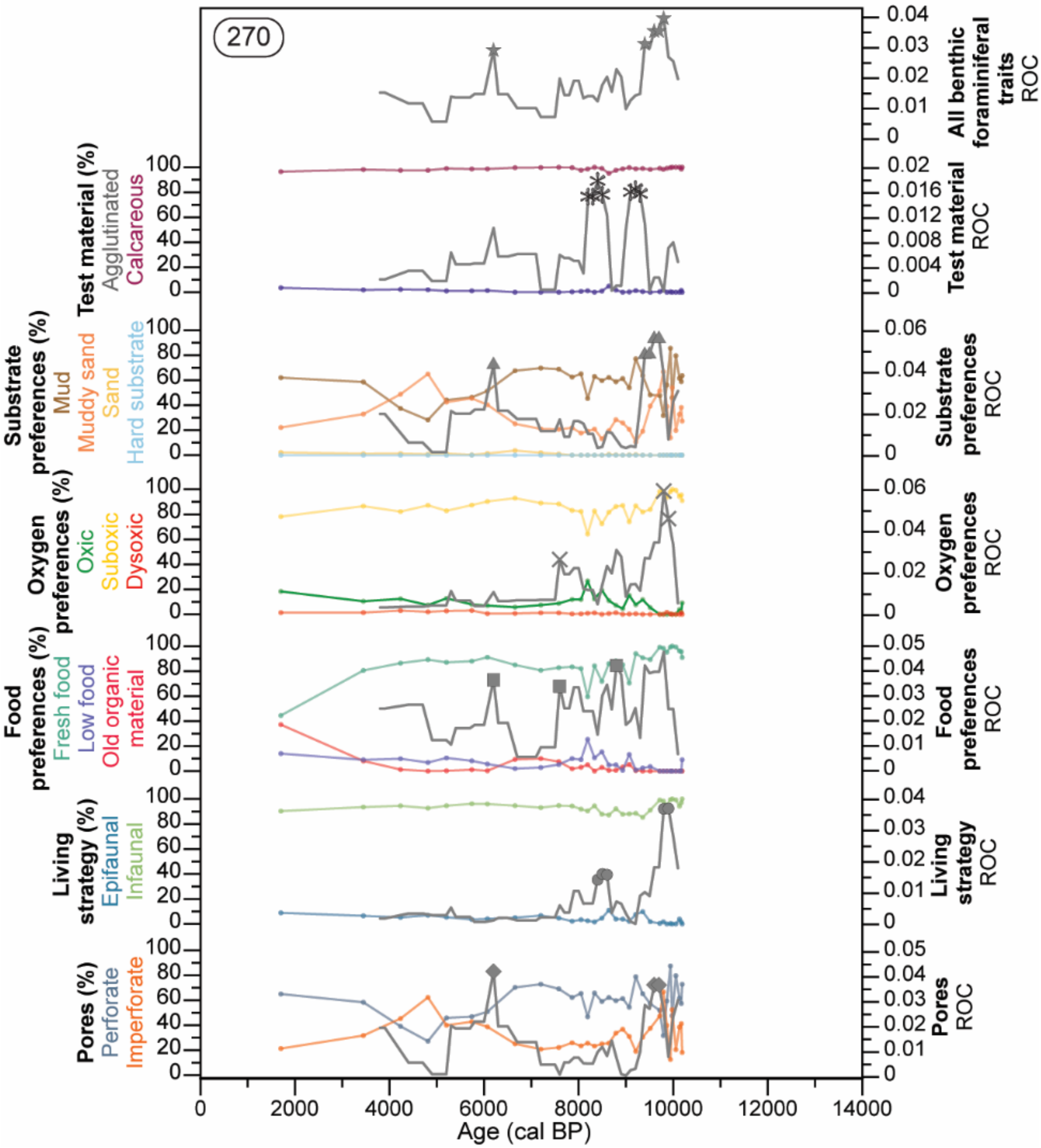

Figure S6: Changes in the benthic foraminiferal traits over time (%) and separate rate-of-change estimates together with significant peak points for each trait for core 270.

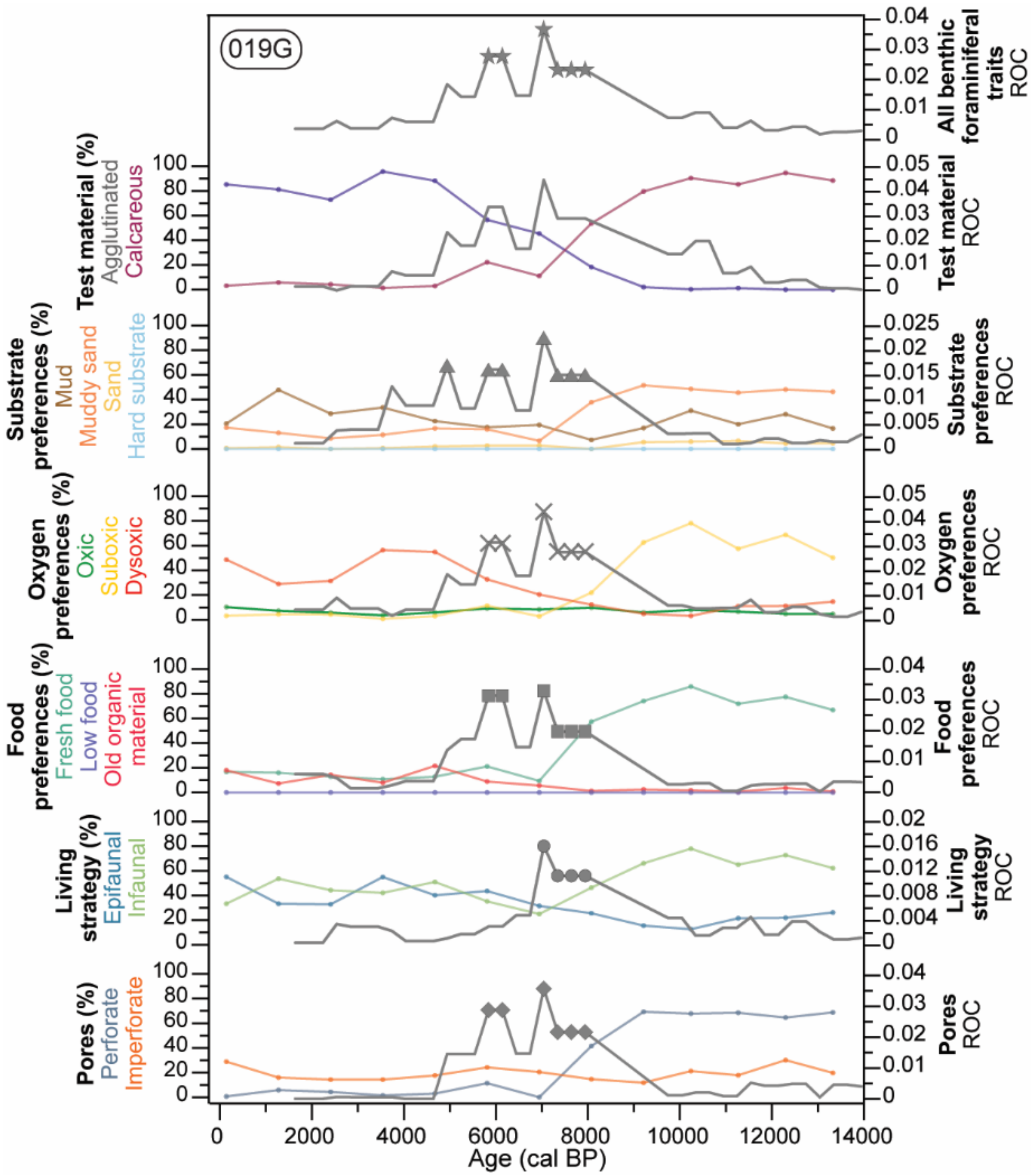

Figure S7: Changes in the benthic foraminiferal traits over time (%) and separate rate-of-change estimates together with significant peak points for each trait for core 019G.

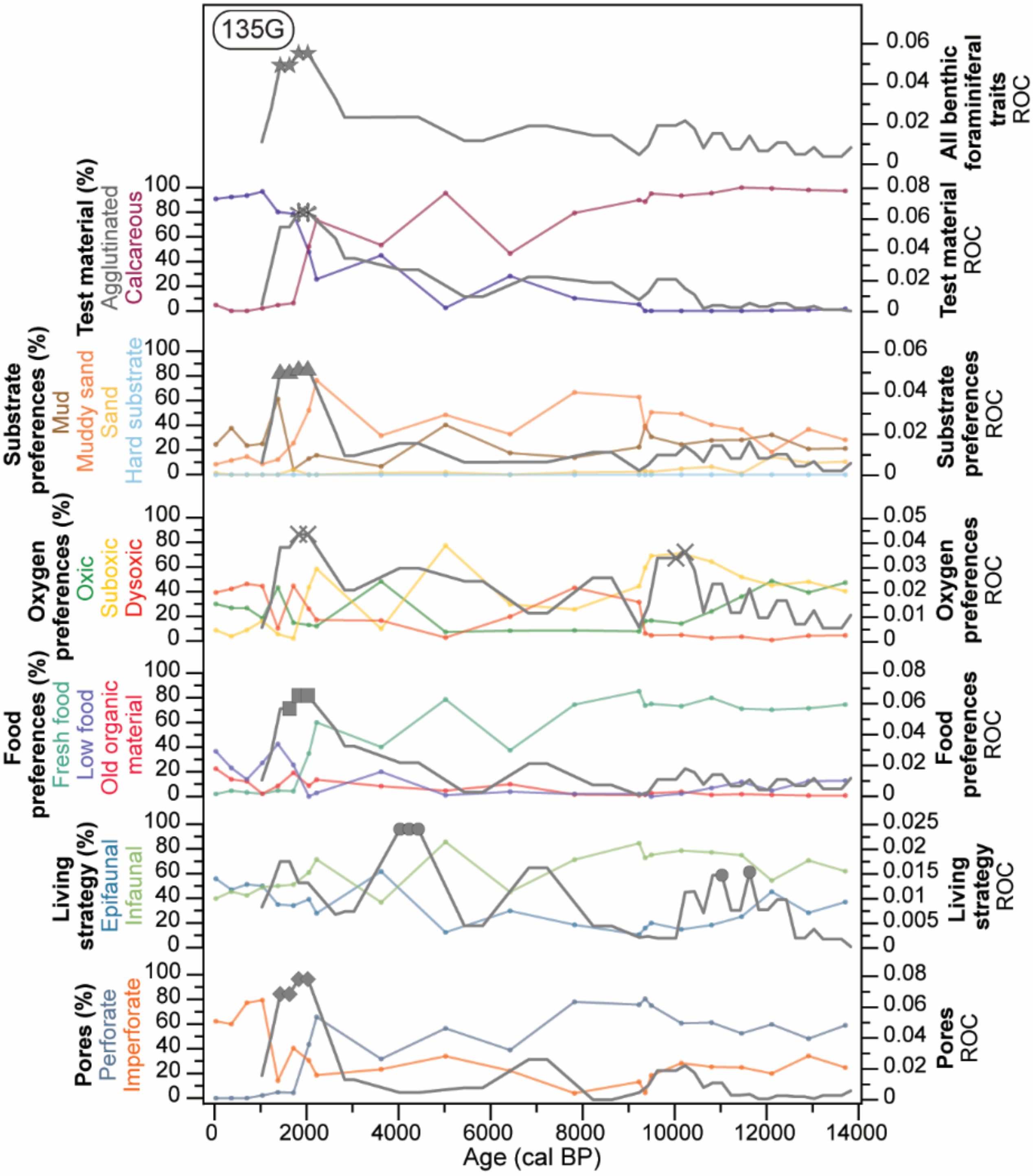

Figure S8: Changes in the benthic foraminiferal traits over time (%) and separate rate-of-change estimates together with significant peak points for each trait for core 135G.

References

1. Wood, S. N. Fast stable restricted maximum likelihood and marginal likelihood estimation of semiparametric generalized linear models. *J. R. Stat. Soc. Ser. B (Statistical Methodol.*

73, 3–36 (2011).

2. Mottl, O. *et al.* Rate-of-change analysis in paleoecology revisited: A new approach. *Rev. Palaeobot. Palynol.* **293**, 104483 (2021).
